# Whole-Genome Sequencing Analysis of ST1, ST11, ST17, and ST37 *Clostridioides* *difficile* Lineages from a Korean Hospital

**DOI:** 10.3390/microorganisms14092122

**Published:** 2026-09-21

**Authors:** Seongjin Cho, Young Kyung Lee, Kibum Jeon, Jae-Seok Kim

**Affiliations:** 1 Department of Laboratory Medicine, Hallym University Sacred Heart Hospital, Hallym University College of Medicine, Anyang 14068, Republic of Korea; piabt3000@hallym.or.kr (S.C.); lyoungk@hallym.or.kr (Y.K.L.); 2 Department of Laboratory Medicine, Hallym University Hangang Sacred Heart Hospital, Hallym University College of Medicine, Seoul 07247, Republic of Korea; pourmythe45@hallym.or.kr; 3 Department of Laboratory Medicine, Kangdong Sacred Heart Hospital, Hallym University College of Medicine, Seoul 05355, Republic of Korea

**Keywords:** *Clostridioides difficile*, whole-genome sequencing, virulence factors, molecular epidemiology, genetic variation

## Abstract

*Clostridioides difficile* infection (CDI) is a major cause of antibiotic-associated diarrhea and pseudomembranous colitis and remains one of the most common healthcare-associated infections. *Clostridioides difficile* sequence types 1 and 11 (ST1 and ST11), which belong to multilocus sequence typing (MLST) clades 2 and 5, respectively, are widely recognized as epidemic-associated lineages. Both lineages harbor virulence-associated genetic features, including truncating variants in *tcdC* and carriage of the binary toxin locus (CdtLoc). ST37 (clade 4) has been an epidemic lineage in East Asia, whereas ST17 (clade 1) has been reported as a predominant lineage in the Republic of Korea and Japan. We performed short-read whole-genome sequencing (WGS) on 61 clinical isolates collected at a university-affiliated hospital in the Republic of Korea between February 2016 and April 2019. Long-read sequencing was additionally performed for 24 isolates, generating complete genomes by hybrid assembly. ST17 was the most prevalent sequence type (*n* = 16, 26.2%), followed by ST1 (*n* = 10, 16.4%). ST1 and ST11 isolates harbored the genetic features reported for these lineages, including truncating variants in *tcdC*. Genomic profiling further identified virulence-associated genes and antimicrobial resistance (AMR) determinants in diverse lineages. These findings characterize the genomic features of co-circulating *C. difficile* lineages and reveal the local presence of epidemic-associated strains.

## 1. Introduction

*Clostridioides difficile* is a Gram-positive, obligately anaerobic, spore-forming bacillus that remains a major cause of healthcare-associated diarrhea worldwide [1]. Clinical manifestations of *C. difficile* infection (CDI) range from self-limiting diarrhea to fulminant colitis, with an overall 90-day mortality of approximately 6% [2,3]. Recent meta-analyses have estimated an incidence of 4.26 CDI cases per 1000 admissions [1] and a prevalence of 30% among hospitalized patients with diarrhea [4].

Since the early 2000s, epidemic-associated *C. difficile* ribotypes, particularly ribotype 027/sequence type 1 (RT027/ST1) and ribotype 078/sequence type 11 (RT078/ST11), have caused major outbreaks in North America and Europe and have been associated with severe clinical outcomes [5,6,7,8]. The emergence of these lineages in East Asia was relatively delayed and became prominent only in the mid-2010s [9,10]. These ribotypes are often described as hypervirulent; however, this term remains controversial because virulence is multifactorial and cannot be defined by lineage, toxin-related features, or antimicrobial resistance (AMR) alone [11,12].

The major virulence factors of *C. difficile* are toxin A (TcdA) and toxin B (TcdB), encoded by *tcdA* and *tcdB* within the chromosomal pathogenicity locus (PaLoc, 19.6 kbp) [2]. The PaLoc additionally contains the genes *tcdR*, *tcdC*, and *tcdE*, which encode a positive regulator, a negative regulator, and a holin-like protein, respectively [13,14,15]. The predominant toxin profile is TcdA+/TcdB+, indicating the production of both major toxins [16]. RT018/ST17 strains generally exhibit this toxin profile and are particularly prevalent in the Republic of Korea and Japan [9,10,17].

In addition to the major toxins TcdA and TcdB, some *C. difficile* strains harbor the binary toxin locus (CdtLoc), which encodes *C. difficile* transferase (CDT). The CdtLoc is approximately 6.8 kbp in size and contains the binary toxin genes *cdtA* and *cdtB,* as well as the positive regulator gene *cdtR* [18]. Substantial genetic variation has been reported in the CdtLoc among clinical isolates, with potential implications for CDT-associated virulence [19,20,21].

RT027, a representative ST1 lineage, is characterized by the presence of the CdtLoc and truncating variants in *tcdC* [22,23]. Consistent with the loss of negative regulation associated with *tcdC* variants, RT027/ST1 strains produce 16-fold and 23-fold more TcdA and TcdB than control strains, respectively [5,6]. Similarly, RT078/ST11 shares virulence-associated genetic features with RT027/ST1, including binary toxin genes and truncating variants in *tcdC* [24].

In Asia, another clinically important toxigenic lineage is ST37, which shows a TcdA−/TcdB+ profile resulting from *tcdA* truncation and, unlike ST1 and ST11, lacks the binary toxin locus (CDT−). ST37 is regarded as one of the most successful TcdA−/TcdB+ lineages in Asia [25,26]. This lineage has been associated with severe CDI and multidrug resistance in Asia [25,26,27]. In addition, TcdA−/TcdB+ strains, including RT017/ST37, have been repeatedly reported among CDI patients in the Republic of Korea [9,28].

Fluoroquinolone resistance has contributed to the emergence and international dissemination of several epidemic *C. difficile* lineages [29,30]. Mutations in *gyrA* (p.Thr82Ile and p.Thr82Val) and *gyrB* (p.Asp426Asn and p.Asp426Val) have been associated with fluoroquinolone resistance [31,32]. In RT027/ST1, the *gyrA* mutation encoding GyrA Thr82Ile was present before its intercontinental spread [29].

PCR ribotyping and multilocus sequence typing (MLST) target different genomic regions and are complementary rather than interchangeable [33]. PCR ribotyping differentiates isolates according to length polymorphisms in the 16S–23S rRNA intergenic spacer regions, whereas MLST assigns sequence types from allelic profiles of seven housekeeping genes [34,35]. Although the two typing schemes are often strongly correlated, their correspondence is not strictly one-to-one: RT027, RT018, and RT017 are commonly associated with ST1, ST17, and ST37, respectively, whereas ST11 encompasses several ribotypes, including RT078 and RT126 [33,36].

Whole-genome sequencing (WGS)-based approaches, such as core genome multilocus sequence typing (cgMLST), provide high-resolution strain discrimination for epidemiological investigations [34]. WGS also allows the analysis of toxin loci, AMR determinants, and other lineage-associated genomic features, providing comparisons with genomes from other countries [37]. In addition, hybrid assembly using short-read and long-read sequencing data can generate complete genomes, facilitating phylogenetic analyses and the identification of genetic variation that may be missed in fragmented assemblies from short-read data alone [38].

Although WGS has increasingly been used to study *C. difficile*, genomic data from Korean isolates remain limited [39]. In this study, we investigated phylogenetic relatedness, toxin-locus profiles, and AMR determinants in isolates from a university-affiliated hospital in the Republic of Korea. We focused on ST1 and ST11 because they include ribotypes historically associated with outbreaks and on ST17 and ST37 because of their epidemiological importance in East Asia.

## 2. Materials and Methods

### 2.1. Collection and Identification of C. difficile Isolates

From February 2016 to April 2019, a total of 61 C. difficile isolates were collected from 61 different patients at a university-affiliated hospital in Seoul, Republic of Korea. The bacterial isolate was the unit of genomic analysis. Stool specimens collected from patients with suspected CDI were cultured to recover the isolates. The cultured isolates were stored in skim milk at −70 °C. For routine subculture, a small portion of the frozen stock was scraped from the surface without completely thawing the stock and inoculated onto culture medium. The isolates were subcultured in a BACTRON 300 anaerobic chamber (Sheldon Manufacturing, Cornelius, OR, USA) with a gas mixture of 85% nitrogen, 10% hydrogen, and 5% carbon dioxide. Selective stool culture was performed with chromID *C. difficile* medium (bioMérieux, Marcy l’Étoile, France) under anaerobic conditions at 37 °C. After 24–48 h of incubation, colonies consistent with *C. difficile* were confirmed using the Bruker MALDI Biotyper Sirius (Bruker, Bremen, Germany). The presence of *tcdB* in the isolates was confirmed by single-round conventional PCR using the second-round primer pair (6880F and 7221R) described by Park et al. [40].

### 2.2. DNA Extraction and WGS

Genomic DNA was extracted using the QIAamp DNA Mini Kit (Qiagen, Hilden, Germany), and DNA concentrations were measured with a Quantus fluorometer (Promega, Madison, WI, USA). For short-read sequencing, genomic libraries were prepared using the Nextera DNA Flex Library Prep Kit (Illumina, San Diego, CA, USA) according to the manufacturer’s instructions and sequenced on the Illumina MiSeqDx platform with 2 × 150 bp paired-end reads. Short-read quality control was performed using fastp (v1.0.1) [41], with a minimum Phred quality score of 20, a minimum read length of 50 bp, and automatic adapter detection and removal.

Long-read sequencing was additionally performed for 24 isolates collected between 2017 and August 2018 that were available for further analysis after short-read sequencing. The selection criteria were not predefined before the study, and selection was based on sample availability rather than on specific genomic findings from the short-read analysis. Genomic libraries were prepared using the Ligation Sequencing Kit (SQK-LSK109; Oxford Nanopore Technologies, Oxford, UK) and sequenced on a MinION flow cell (FLO-MIN106, R9.4.1; Oxford Nanopore Technologies). The raw long-read signal data (FAST5) were re-basecalled using Dorado (v0.9.6) with the super-accuracy model to improve basecalling accuracy. Sequencing adapters were trimmed using the default settings. Long-read quality filtering was performed using Filtlong (v0.3.1) [42], retaining reads of at least 1000 bp and the highest-quality 95% of reads. When available, the corresponding Illumina short reads were used as external references to guide Filtlong quality estimation.

### 2.3. Genome Assembly and Annotation

We performed de novo assembly of Illumina short-read sequences from all 61 isolates using Unicycler (v0.5.1) [43] in short-read-only mode, with the normal bridging mode, a minimum FASTA contig length of 100 bp, and a depth-filter threshold of 0.25 of the chromosomal depth.

For the 24 selected isolates, long-read consensus assemblies were generated using Autocycler (v0.5.2) [44] with the autocycler_full.sh workflow and subsequently polished with the corresponding Illumina short reads to produce hybrid assemblies. Autocycler was used to integrate the outputs of multiple long-read assemblers; the filtered long reads were subsampled into four sets, assembled independently by multiple long-read assemblers. The resulting assemblies were compressed, clustered, trimmed, resolved, and combined into a consensus assembly. Replicon circularity was determined from the topology of the final consensus assembly graph, and an assembly was considered fully resolved when all replicons were represented as single circular contigs. The resulting consensus assemblies were polished first with the long reads using Medaka (v2.1.1) [45] and subsequently with the corresponding Illumina short reads using Polypolish (v0.6.1) [46] and pypolca (v0.4.0) [47].

Assembly quality was assessed using QUAST (v5.3.0) [48], and genome completeness was evaluated using BUSCO (v6.0.0) [49] with the peptostreptococcaceae_odb12 dataset. The short-read-only and hybrid assemblies were annotated using Prokka (v1.14.6) [50] and Bakta (v1.12.0) [51], respectively. Prokka was run with bacterial genetic code 11 and a minimum contig length of 200 bp. The GenBank file for strain 630 was supplied as a reference. Bakta was run with the full database v6.0 (release date: 24 February 2025), a minimum contig length of 200 bp, and the complete-genome option.

All non-chromosomal circular contigs from the 24 hybrid assemblies were analyzed as extrachromosomal elements (ECEs). Plasmid replicons were assessed using MOB-suite (v3.1.9) [52] and PlasmidFinder (v2.1.6) [53], and sequence similarity to reference genomes was assessed using BLASTn (BLAST+ v2.17.0) [54]. ECEs containing all four structural components of the phage virion (capsid, portal, terminase, and tail) and lacking plasmid replication genes were classified as extrachromosomal phages. ECEs containing at least three of the four core virion modules and plasmid replication genes were classified as putative phage-plasmids. ECEs with fewer than three core virion modules were classified as plasmids if they contained plasmid replication genes or were similar to a previously reported *C. difficile* plasmid. All other parameters not specified here were left at their defaults.

We additionally retrieved a curated collection of 34 complete *C. difficile* genomes from the National Center for Biotechnology Information (NCBI) database, using the NCBI Datasets tool to download RefSeq complete-genome metadata (accessed on 15 April 2026). Eligibility was based on the RefSeq assembly-level designation “Complete Genome”. Records were included when the BioSample metadata indicated both Korean origin (“South Korea,” “Republic of Korea,” or “Korea”) and human origin (“Homo sapiens” or “Human”); records not meeting both criteria were excluded. The assemblies were used as deposited, without reannotation or polishing. The accession numbers of all 34 assemblies are provided in Table S1.

### 2.4. Genome Alignment and Phylogenetic Analysis

Whole-genome alignment of the 24 hybrid assemblies was performed using Mauve (v2.4.0) [55] to assess genomic structures. Sequence types were determined using MLST (v2.23.0) [56]. MLST clades were assigned according to the established *C. difficile* clade classification [57].

A cgMLST schema was constructed from the 24 hybrid assemblies using chewBBACA (v3.5.3) [58], and allelic profiles were assigned to all 61 isolates. A minimum spanning tree based on the allelic distances was generated using GrapeTree v2.2 [59] and visualized in iTOL v7 [60].

### 2.5. Variant Detection and Toxin Locus Visualization

Single nucleotide variants (SNVs) and short insertions and deletions (indels) were detected from the short-read sequencing data using Snippy (v4.6.0) [61], with *C. difficile* strain 630 (accession number NC_009089.1) as the primary reference genome.

For the CdtLoc region, strain CD196 (accession number NC_013315.1) was used as the reference because strain 630 lacks the CdtLoc. In the standard Snippy workflow, variant effects were annotated using SnpEff (v5.0e) [62]. Variants predicted to result in stop-gained, stop-lost, start-lost, frameshift, or disruptive in-frame alterations were categorized as high impact. We screened variants in the PaLoc, CdtLoc, and functional genes associated with sporulation, adhesion, motility, and stress response. We also screened for the established fluoroquinolone resistance-associated substitutions *gyrA* p.Thr82Ile and p.Thr82Val, and *gyrB* p.Asp426Asn and p.Asp426Val. The structural organization of the toxin loci was visualized using pyGenomeViz (v1.6.1) [63].

### 2.6. Identification of Virulence and AMR Genes

Using ABRicate (v1.2.0) [64], virulence genes were screened against the Virulence Factor Database (VFDB) [65], and AMR genes were screened against the Comprehensive Antibiotic Resistance Database (CARD) [66] and ResFinder [67]. A minimum threshold of 80% was applied to both sequence identity and coverage.

### 2.7. Statistical Analysis

All statistical analyses were performed using R version 4.5.2 (R Foundation for Statistical Computing, Vienna, Austria) and SPSS Statistics version 30.0 (IBM Corp., Armonk, NY, USA).

## 3. Results

### 3.1. Genomic Characteristics of Short-Read Assemblies and Hybrid Assemblies

The draft genome assemblies generated using only Illumina reads from all 61 isolates had total lengths of 3.94–4.37 Mbp (mean 4.17 Mbp) and remained fragmented, with a median of 95 contigs (range 54–160). For 24 of the 61 isolates, long-read sequencing data were additionally incorporated to generate hybrid assemblies (Table 1). All 24 isolates subjected to hybrid assembly yielded complete genome assemblies with improved contiguity. These complete genome assemblies ranged from 4.04 to 4.46 Mbp in total length (mean 4.24 Mbp). The complete genomes contained 0–2 circular ECEs each. In total, 24 ECEs ranging from 6.7 to 134.7 kbp were identified in 18 of the 24 isolates, with the remaining six carrying no ECEs (Table S8).

Publicly available complete genome assemblies of *C. difficile* isolates collected in the Republic of Korea were retrieved and analyzed separately to characterize their genomic features. The characteristics of these genomes are summarized in Table S1. The public dataset comprised 34 complete genomes and was dominated by ST203 (*n* = 14), ST42 (*n* = 12), and ST129 (*n* = 6); ST17 and an isolate with an unassigned sequence type were each represented by a single genome.

### 3.2. Structural Comparison of 24 Complete C. difficile Genomes

Alignment of the 24 hybrid assemblies revealed multiple locally collinear blocks shared across the isolates (Figure S1). A subset of genomes showed differences in the order or orientation of these blocks, consistent with structural rearrangements among isolates.

### 3.3. Phylogenetic Relationships

Of the 61 isolates, 60 were assigned to four MLST clades. Clade 1 predominated (*n* = 45, 73.8%) and included 14 STs, followed by clade 2 (ST1; *n* = 10, 16.4%), clade 4 (ST37; *n* = 3, 4.9%), and clade 5 (ST11; *n* = 2, 3.3%). The remaining isolate had an unassigned ST. At the ST level, ST17 (*n* = 16, 26.2%) was the most prevalent, followed by ST1 (*n* = 10, 16.4%); the complete distribution is provided in Table S2.

**Figure 1:**
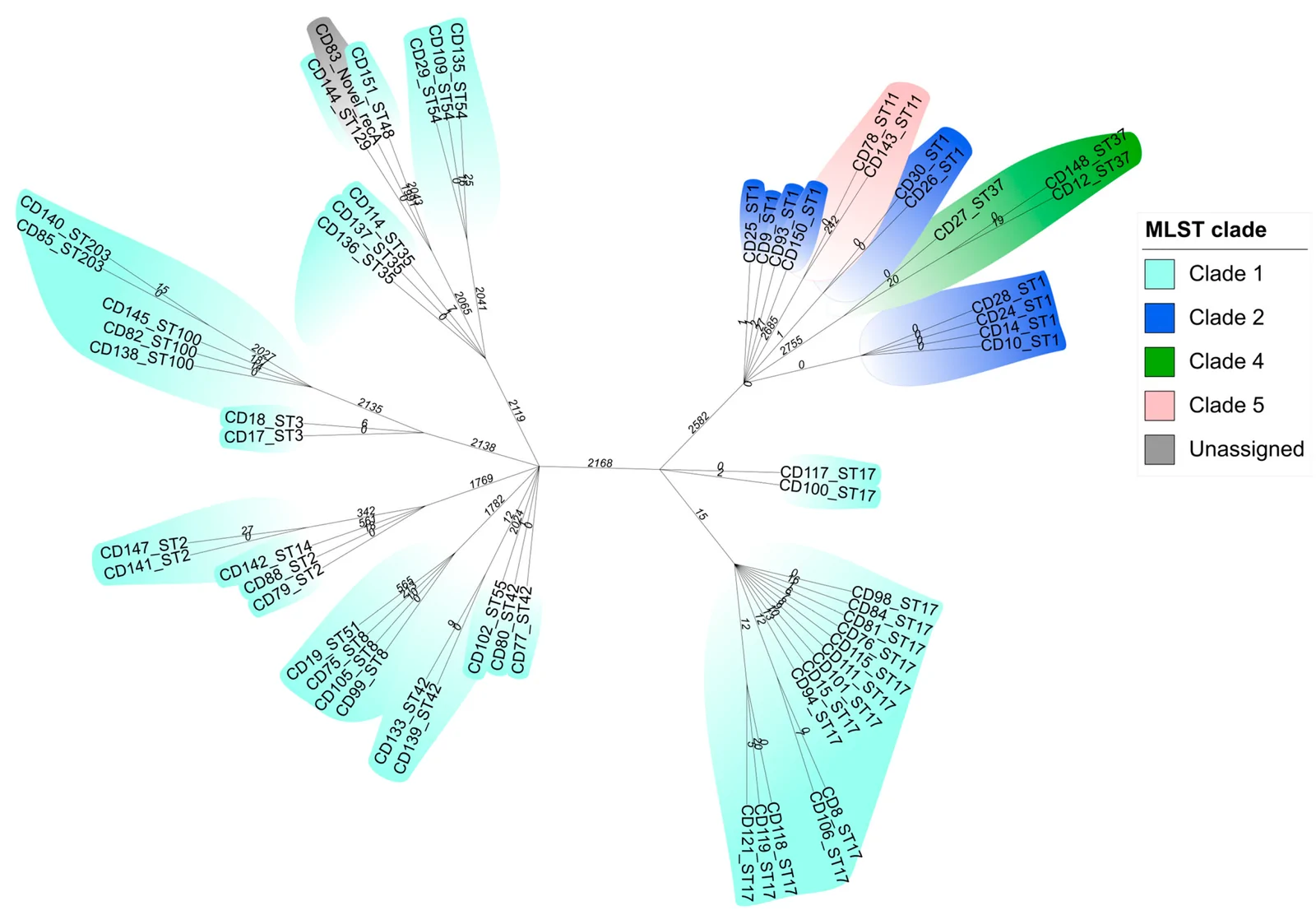
Minimum spanning tree based on cgMLST analysis. Each node is labeled with the isolate identifier, and the numeric labels on the connecting branches represent allelic differences between the linked isolates. Clades 1, 2, 4, and 5 are shown in distinct colors, whereas the unassigned isolate is shown in gray. Isolates clustered according to sequence type, and the sequence-type clusters were nested within their respective clades. ST1 (clade 2) and ST17 (clade 1) formed three and four subclusters, respectively. ST37 (clade 4) comprised a single isolate and a two-isolate cluster. The two ST11 isolates (clade 5) exhibited the greatest within-lineage allelic difference.

Phylogenetic analysis revealed clustering by sequence type, and the ST-specific clusters nested within their corresponding MLST clades (Figure 1). ST1 (clade 2), ST11 (clade 5), ST17 (clade 1), and ST37 (clade 4) formed separate groups in the minimum spanning tree. Allelic differences among isolates were small within each sequence type, whereas the two ST11 isolates showed the greatest within-lineage genetic distance.

### 3.4. Genetic Variants in the PaLoc and CdtLoc Regions of ST1 and ST11

**Figure 2:**
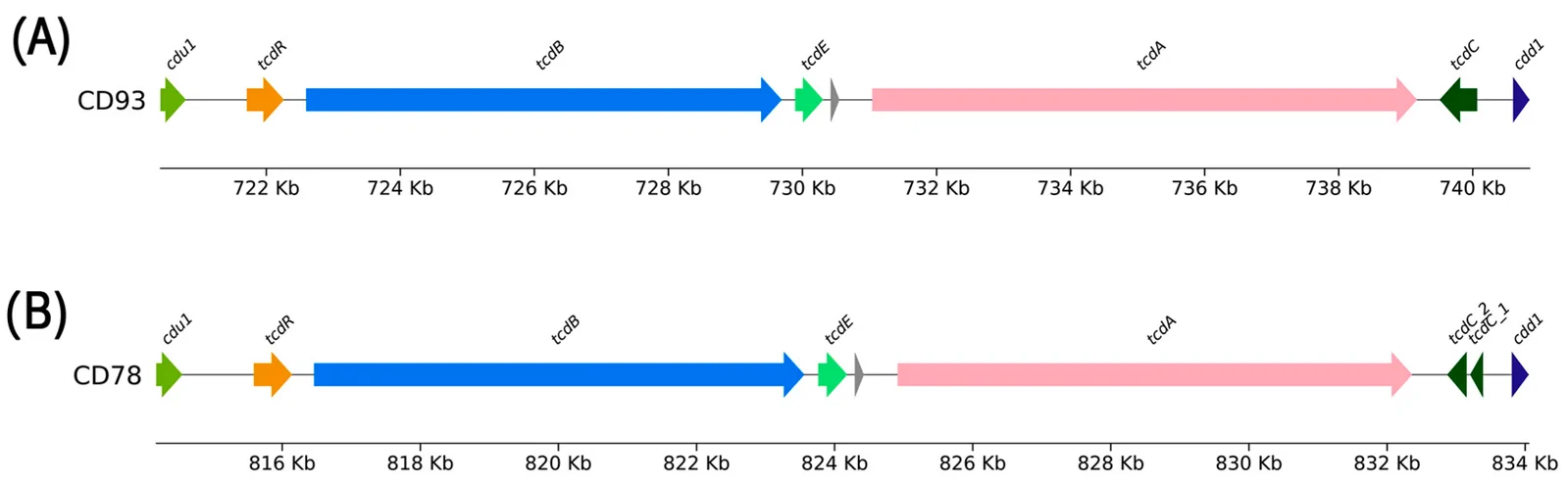
Schematic diagram of the PaLoc region and adjacent genes in ST1 and ST11 lineages. Only complete genome assemblies were included. Arrows represent genes, and arrowheads indicate transcriptional orientation. (**A**) ST1 isolate CD93 retains intact *tcdA* and *tcdB*. A frameshift variant in *tcdC* is predicted to truncate *tcdC*. (**B**) ST11 isolate CD78 retains an intact *tcdB* allele but carries a shortened *tcdA* allele of 7452 bp. Two stop-gained variants in *tcdC* are predicted to divide the gene into two fragments.

Multiple structural alterations and sequence variants were detected in the PaLoc region among the epidemic-associated ST1 and ST11 isolates (Table 2, Figure 2). All ST1 isolates (*n* = 10) harbored a frameshift mutation in *tcdC* (p.Gly40Valfs*27), and isolate CD10 additionally carried an in-frame deletion (p.Ala111_Lys116del). ST11 isolates (*n* = 2) carried two nonsense mutations in *tcdC* (p.Gln62* and p.Tyr186*) and shortened *tcdA* alleles (7452 bp in CD78 and 291 bp in CD143), compared with the 8133 bp *tcdA* allele of the reference strain 630.

High-impact CdtLoc variants of ST1 and ST11 isolates are summarized in Table S3. The CdtLoc was conserved among the ST1 isolates (*n* = 10), with no high-impact variants detected. In one of the two ST11 isolates (CD143), a stop-gained variant (p.Glu108*) was detected in *cdtR*.

### 3.5. Virulence Factor Gene Profiles

A total of 12 virulence factor genes were identified. These genes were classified into four functional categories: (1) toxin genes (*tcdA* and *tcdB*), (2) adhesion-associated genes (*fbpA*, *CD0873*, *cbpA*, *CD2831*, and *groEL*), (3) cell surface structure components (*cwp66*, *CD3246*, *cwp84*, and *slpA*), and (4) the zinc metalloprotease gene *zmp1*. Virulence gene profiles are summarized in Table S4.

All ST1 isolates (*n* = 10) harbored *tcdA*, *tcdB*, *fbpA*, *CD0873*, *CD2831*, *cwp66*, *cwp84*, *zmp1*, and *groEL*. Among ST11 isolates (*n* = 2), both harbored *tcdB*, *fbpA*, *CD0873*, *CD2831*, *CD3246*, *cwp84*, *zmp1*, and *groEL*, while *tcdA* was not called by the virulence gene screening because the truncated alleles were not detected with the applied screening threshold; *cbpA* was detected only in CD78 and *cwp66* only in CD143. ST37 isolates (*n* = 3) harbored all genes except *tcdA*. In ST17 isolates (*n* = 16), all screened genes except *slpA* were called.

### 3.6. AMR Gene Profiles

The distribution of AMR genes is summarized in Table S5. Among ST1 isolates (*n* = 10), all carried *CDD-2*, *cplR*, *nimB*, and *cdeA*, and 9 of 10 isolates (90.0%) harbored *cfrC* and *ermB*. ST1 isolates lacked *aac(6′)-aph(2″)* and tetracycline resistance genes.

ST11 isolates (*n* = 2) carried *CDD-1*, *cplR*, *nimB*, *cdeA*, and *tet(M)*. One ST11 isolate (CD143) additionally carried *aac(6′)-aph(2″)*, *tet(40)*, and *tet(O/M/O)*.

Among ST17 isolates (*n* = 16), all harbored *CDD-1*, *cplR*, *nimB*, *ermB*, and *cdeA*, and 15 of 16 (93.8%) harbored *aac(6′)-aph(2″)*. None of the ST17 isolates carried *CDD-2*, *cfrC*, or tetracycline resistance genes.

ST37 isolates (*n* = 3) exhibited a distinct resistance gene profile. *CDD-2*, *aac(6′)-aph(2″)*, *cplR*, *nimB*, *ermB*, *cdeA*, and *tet(M)* were present in all isolates. In addition, *cfrC* and *erm(52)* were identified in CD27, and *dfrF* was detected in CD148. ST37 isolates lacked *CDD-1*, *tet(40)*, and *tet(O/M/O)*.

Fluoroquinolone resistance-associated determinants were identified in all four lineages (Table S6). *gyrA* p.Thr82Ile was detected in 9 of 10 ST1 isolates and all ST17 and ST37 isolates, whereas *gyrB* p.Asp426Asn was detected in one ST11 isolate.

### 3.7. Variants in Genes with Functional Relevance

Multiple high-impact variants were detected in six genes with functional relevance: *bclA1*, *slpA*, *cbpA*, *flgL*, *fliS*, and *uvrA* (Table S7).

All ST1 isolates (*n* = 10) exhibited sequence variants in sporulation- and motility-related genes: *bclA1* with two premature stop codons (p.Lys49* and p.Gln574*), *flgL* with an in-frame deletion (p.Asn218del), and *fliS* with a stop-lost variant (p.Ter117LysextTer2).

ST11 isolates (*n* = 2; CD78 and CD143) carried four disruptive in-frame variants in *cbpA* (p.Gly963_Asp969delinsProAsn, p.Lys713delinsAsnTyr, p.Gly637del, and p.Asp605delinsGluIleAsn). They also carried a disruptive in-frame deletion in *flgL* (p.Asn218del) and a stop-lost variant in *fliS* (p.Leu116_Ter117delinsIleLysIleTer). Additionally, CD143 harbored a disruptive in-frame deletion in *slpA* (p.Glu634_Val635delinsAla), and CD78 harbored a frameshift variant in *uvrA* (p.Gln703Thrfs*12).

All ST17 isolates (*n* = 16) harbored a disruptive in-frame deletion in *slpA* (p.Glu634_Val635delinsAla) and a stop-lost variant in *fliS* (p.Ter117LysextTer2). A disruptive in-frame deletion in *flgL* (p.Asn218del) was observed in 3 of 16 isolates.

Among the ST37 isolates, two of three carried a disruptive in-frame insertion in *cbpA* (p.Asp1069_Ile1070insIleAsp), and no high-impact variants were identified in other genes.

## 4. Discussion

WGS facilitates a comprehensive understanding of regional CDI epidemiology by delineating lineage diversity. Effective CDI management requires distinguishing endemic spread from the introduction of epidemic-associated lineages. In this study, integrated genomic analysis of 61 clinical isolates from a Korean hospital revealed the co-circulation of clade 2 (ST1) and clade 5 (ST11) lineages with locally prevalent clade 1 (ST17) and clade 4 (ST37) lineages.

We generated Illumina-based genome assemblies for all 61 isolates and reconstructed 24 complete genomes using hybrid assembly. Because hybrid assembly requires more sequencing resources than short-read sequencing alone, we performed long-read sequencing on a subset of isolates and retained Illumina-based assemblies for phylogenetic and gene content analyses across the entire cohort.

The MLST clade assignments place the observed ST diversity within the established phylogenetic framework of *C. difficile* [68,69]. Among the study isolates, the majority of isolates clustered in clade 1, which was genetically heterogeneous, whereas clades 2, 4, and 5 comprised ST1, ST37, and ST11 isolates, respectively. This clade-level distribution is epidemiologically relevant because these clades differ in toxin locus architecture, host associations, and AMR profiles [30,36,69].

We focused on ST1, ST11, ST17, and ST37 lineages among our isolates. ST1 belongs to clade 2 and is strongly associated with RT027, whereas ST11 belongs to clade 5 and encompasses several epidemiologically important ribotypes, including RT078 and RT126 [70]. RT018/ST17 belongs to the genetically heterogeneous clade 1, and RT017/ST37 belongs to clade 4 [70]. A large clinical study of 2222 cases reported that 14-day mortality was 25% in clade 5 (RT078/ST11), 20% in clade 2 (predominantly RT027/ST1), and 14% in clade 4 (predominantly RT017/ST37) [71].

RT027/ST1 and RT078/ST11 were rare in Korean datasets from the 2010s, each accounting for only 0 to 0.9% of the total isolates [9,28,72]. Similarly, a recent analysis from the Republic of Korea [73] reported no ST1 isolates and a low prevalence of ST11 (*n* = 4, 1.8%). We observed higher frequencies of ST1 (*n* = 10, 16.4%) and ST11 (*n* = 2, 3.3%) in our clinical isolates. However, the inference that this represents an epidemiological shift requires more support than a comparison of proportions between studies; therefore, our findings should be interpreted as evidence of the local presence of ST1 and ST11 in this study population rather than as evidence of a temporal epidemiological shift.

Although ST1, ST11, ST17, and ST37 are commonly referred to as clonal lineages, the within-ST diversity observed by cgMLST shows that shared sequence type alone cannot establish transmission between cases. Genomic surveillance for nosocomial transmission therefore requires cgMLST and SNP-level resolution. Within-lineage genetic heterogeneity of this kind has been documented for each of the lineages [25,36,74,75,76], and the pattern we observed is most consistent with repeated introduction of distinct clones from the wider community or referral network, followed by limited local spread, rather than with sustained single-clone transmission.

TcdC is an anti-sigma factor that represses transcription of *tcdA* and *tcdB* by antagonizing TcdR, so truncation of *tcdC* has been proposed to relieve repression and thereby increase toxin production [13,14,15,77]. All 10 of our ST1 isolates carried the *tcdC* frameshift variant c.117_120delinsGGT (p.Gly40Valfs*27). Isolate CD10 additionally carried the 18 bp in-frame deletion c.330_347del (p.Ala111_Lys116del), corresponding to the deletion pattern described in RT027 [22,77,78]. Both ST11 isolates carried c.183_184delinsTT (p.Gln62*), corresponding to the C184T nonsense mutation characteristic of RT078/ST11 [24,79], although this variant is not RT078-specific and has been reported in RT066/ST11 and in several unassigned non-078 ribotypes [23,79,80].

The two ST11 isolates showed apparently shortened *tcdA* alleles, measuring 7452 bp in CD78 and 291 bp in CD143. Because *tcdA* contains an extensive region of combined repetitive oligopeptides, short-read assemblies may collapse repeat copies and consequently underestimate the allele length [16]. In particular, the extremely short 291 bp *tcdA* sequence identified in CD143 should be interpreted with caution, because it may reflect incomplete assembly or annotation rather than the true length of the gene.

ABRicate screening revealed a widespread distribution of virulence-associated genes outside the PaLoc. Ten genes were identified, comprising five adhesion-related genes (*fbpA*, *CD0873*, *cbpA*, *CD2831*, and *groEL*), four genes encoding cell surface proteins (*cwp66*, *CD3246*, *cwp84*, and *slpA*), and the zinc metalloprotease gene *zmp1*. Cwp66 contributes to cell adhesion and stress tolerance [81], CbpA is a surface-exposed adhesin that binds to human collagens I and V [82], and CD3246 is a surface protein associated with biofilm formation [26,83]. Although most of the virulence-associated genes were broadly distributed, the detection frequency of *cwp66*, *cbpA*, and *CD3246* differed among ST1, ST11, ST17, and ST37 isolates. These lineage-associated differences suggest that variation in surface and adhesion-related factors may influence colonization, persistence, and host interaction independently of the major toxin loci. However, in the absence of functional assays and linked clinical outcomes, these genomic differences should be regarded as candidate lineage-associated traits rather than evidence of altered virulence.

The AMR-associated genes *nimB*, *cplR*, and *cdeA* were detected in all 61 isolates and are also widespread across the *C. difficile* species [84]. Because *nimB* is nearly universal yet its presence alone does not confer metronidazole resistance, its detection should not be interpreted as predictive of phenotypic resistance [85]. In contrast, *ermB* was present in all ST17 and ST37 isolates and in 9 of 10 ST1 isolates, consistent with the phenotypic erythromycin and clindamycin resistance documented in RT018/ST17 and RT017/ST37 [25,86,87]. Regarding fluoroquinolone resistance, the substitutions *gyrA* p.Thr82Ile and *gyrB* p.Asp426Asn were identified. In particular, *gyrA* p.Thr82Ile has been associated with the emergence and dissemination of epidemic *C. difficile* lineages [29,30,32].

This study has several limitations. Isolates were collected from a single center over a relatively short period, and the findings may not reflect the epidemiology of *C. difficile* lineages in the Republic of Korea. Antimicrobial susceptibility testing and phenotypic assays of virulence were not performed. Therefore, the AMR determinants and virulence-associated features identified in this study could not be directly linked to their corresponding phenotypes. In addition, detailed clinical metadata were unavailable, precluding direct evaluation of associations between specific genomic features and clinical outcomes.

## 5. Conclusions

This study provides a genomic framework for understanding diverse *C*. *difficile* lineages circulating in a Korean hospital, including epidemic-associated ST1 (clade 2) and ST11 (clade 5) and regionally important ST17 (clade 1) and ST37 (clade 4). The complete genome assemblies generated in this study expand the genomic resources available for Korean isolates. Multicenter surveillance incorporating WGS, phenotypic antimicrobial susceptibility testing, and linked clinical data is needed to determine whether the observed frequencies of clade 2 and clade 5 lineages reflect broader epidemiological patterns in the Republic of Korea and whether particular lineages or genomic features are associated with clinical outcomes. Toxin quantification and functional studies are also required before variants in *tcdC*, *cdtR*, or other toxin-locus genes can be linked to altered toxin production and virulence.

## Figures and Tables

**Table 1:** Genomic characteristics of *C. difficile* isolates based on short-read-only and hybrid genome assemblies.

Sample ID	Sequence Type	MLST Clade	Completeness (%)	Number of Contigs	Total Length (bp)	Number of ECEs	ECE Length (bp)
CD75 *	ST8	1	100	3	4,242,194	2	129,338 ^a^/46,315 ^a^
CD76 *	ST17	1	99.8	3	4,399,472	2	34,286 ^b^/7639
CD77 *	ST42	1	100	2	4,165,201	1	32,787 ^b^
CD78 *	ST11	5	100	1	4,134,548	0	0
CD79 *	ST2	1	99.8	2	4,193,697	1	7180
CD80 *	ST42	1	100	2	4,165,175	1	32,786 ^b^
CD81 *	ST17	1	99.8	2	4,351,584	1	33,486 ^b^
CD82 *	ST100	1	100	3	4,118,775	2	110,291 ^a^/8104
CD83 *	NA	NA	99.8	2	4,094,353	1	6829
CD84 *	ST17	1	99.8	2	4,341,723	1	39,279 ^b^
CD85 *	ST203	1	99.8	2	4,385,859	1	33,482 ^b^
CD88 *	ST2	1	99.8	1	4,092,908	0	0
CD93 *	ST1	2	99.8	2	4,041,594	1	6670
CD94 *	ST17	1	99.8	2	4,335,854	1	33,486 ^b^
CD98 *	ST17	1	99.8	2	4,351,843	1	33,486 ^b^
CD99 *	ST8	1	99.8	1	4,126,718	0	0
CD100 *	ST17	1	99.8	3	4,294,295	2	33,486 ^b^/8329
CD101 *	ST17	1	100	1	4,095,676	0	0
CD102 *	ST55	1	100	1	4,221,975	0	0
CD105 *	ST8	1	100	2	4,146,830	1	46,181 ^a^
CD106 *	ST17	1	99.8	3	4,331,376	2	33,486 ^b^/8041
CD109 *	ST54	1	99.8	3	4,345,282	2	34,287 ^b^/7793
CD111 *	ST17	1	99.8	1	4,431,866	0	0
CD114 *	ST35	1	99.5	2	4,455,419	1	134,676 ^b^
CD8	ST17	1	99.8	97	4,314,859	NA	NA
CD9	ST1	2	100	121	4,138,630	NA	NA
CD10	ST1	2	100	119	4,132,888	NA	NA
CD12	ST37	4	100	83	4,373,820	NA	NA
CD14	ST1	2	100	121	4,135,157	NA	NA
CD15	ST17	1	99.8	130	4,331,192	NA	NA
CD17	ST3	1	99.5	83	4,050,944	NA	NA
CD18	ST3	1	99.5	77	4,050,780	NA	NA
CD19	ST51	1	100	81	4,054,343	NA	NA
CD24	ST1	2	100	117	4,133,663	NA	NA
CD25	ST1	2	100	119	4,133,067	NA	NA
CD26	ST1	2	100	116	4,129,372	NA	NA
CD27	ST37	4	100	76	4,359,287	NA	NA
CD28	ST1	2	100	123	4,133,151	NA	NA
CD29	ST54	1	99.8	80	4,217,856	NA	NA
CD30	ST1	2	100	115	4,130,744	NA	NA
CD115	ST17	1	99.8	81	4,278,036	NA	NA
CD117	ST17	1	99.8	78	4,227,574	NA	NA
CD118	ST17	1	99.8	138	4,319,243	NA	NA
CD119	ST17	1	99.5	112	4,315,041	NA	NA
CD121	ST17	1	99.8	101	4,316,016	NA	NA
CD133	ST42	1	100	77	4,013,453	NA	NA
CD135	ST54	1	99.8	102	4,262,729	NA	NA
CD136	ST35	1	99.5	135	4,224,539	NA	NA
CD137	ST35	1	99.5	132	4,226,538	NA	NA
CD138	ST100	1	100	87	4,165,994	NA	NA
CD139	ST42	1	100	73	4,020,651	NA	NA
CD140	ST203	1	99.8	108	3,963,737	NA	NA
CD141	ST2	1	99.8	106	4,187,512	NA	NA
CD142	ST14	1	99.8	140	4,170,030	NA	NA
CD143	ST11	5	100	98	3,938,316	NA	NA
CD144	ST129	1	99.8	73	4,102,786	NA	NA
CD145	ST100	1	100	72	4,098,284	NA	NA
CD147	ST2	1	99.8	92	4,206,392	NA	NA
CD148	ST37	4	100	87	4,274,811	NA	NA
CD150	ST1	2	100	146	4,153,213	NA	NA
CD151	ST48	1	99.8	110	4,145,799	NA	NA

NA: Not available; ECE: Extrachromosomal element; Completeness was estimated using BUSCO v6.0.0. * For these isolates, complete genomes were obtained by additional long-read sequencing and hybrid assembly, and the metrics are from the hybrid assemblies; ^a^ Putative phage-plasmid; ^b^ Extrachromosomal phage; unmarked ECEs were classified as plasmids.

**Table 2:** High-impact PaLoc variants among ST1 and ST11 isolates.

Sequence Type	Sample ID	*tcdC*			
		c.117_120delinsGGT p.Gly40Valfs*27	c.330_347del p.Ala111_Lys116del	c.183_184delinsTT p.Gln62*	c.558T > A p.Tyr186*
ST1	CD9/14/24/25/26/28/30/93/150 (*n* = 9)	+			
ST1	CD10 (*n* = 1)	+	+		
ST11	CD78/143 (*n* = 2)			+	+

The asterisk (*) denotes a stop codon; the plus sign (+) indicates the presence of the corresponding variant.

## Data Availability

The original contributions presented in this study are included in the article/Supplementary Material. Further inquiries can be directed to the corresponding author. All 24 complete hybrid genome assemblies presented in this study are publicly available in the NCBI GenBank database at https://www.ncbi.nlm.nih.gov/bioproject/PRJNA1512160 (accessed on 17 September 2026), under BioProject number PRJNA1512160.

## Supplementary Materials

The following supporting information can be downloaded at https://www.mdpi.com/article/10.3390/microorganisms14092122/s1. Table S1: Metadata summary and accession numbers of publicly available complete genomes from Korea; Table S2: Distribution of sequence types and MLST clades in 61 *C. difficile* isolates; Table S3: High-impact CdtLoc variants among ST1 and ST11 isolates; Table S4: Distribution of virulence-associated genes in *C. difficile* isolates; Table S5: AMR gene profiles of *C. difficile* isolates; Table S6: Fluoroquinolone resistance-associated *gyrA* and *gyrB* substitution profiles of *C. difficile* isolates; Table S7: High-impact variants in functional genes summarized for ST1, ST11, ST17, and ST37 isolates; Table S8: Genomic characteristics and classification of the 24 extrachromosomal elements (ECEs); Figure S1: Whole-genome alignment of the 24 hybrid assemblies of *C. difficile* isolates using Mauve (v2.4.0). Colored blocks represent shared homologous regions, and connecting lines mark aligned segments.
