# Supplementary material for: Whole-Genome Sequencing Analysis of ST1, ST11, ST17, and ST37 Clostridioides difficile Lineages from a Korean Hospital
Source: Microorganisms. 2026 Sep 21;14(9):2122. doi: 10.3390/microorganisms14092122 (PMC13609255; doi:10.3390/microorganisms14092122)
Supplement: Supplementary file 1 [file microorganisms-14-02122-s001.zip › microorganisms-4513909-supplementary.pdf]

Supplementary Materials

Table S1. Metadata summary and accession numbers of publicly available complete genomes from Korea.

Strain	Accession Number	Submitter	Collection Date	Sequence Type	Number of Contigs	Total Length (bp)	GC (%)	N50 (bp)	Coverage
BR81	GCF_002007885.1	Kyung Hee University	2016-04-01	ST42	1	4,124,384	28.5	4,124,384	275x
CBA7204	GCF_009867095.1	World Institute of Kimchi	2017-09-26	ST203	1	4,038,993	28.5	4,038,993	240x
Z1322HCD0002	GCF_037942165.1	Jeonbuk National University	2022-07-18	ST203	1	4,067,876	28.5	4,067,876	149x
Z1322HCD0004	GCF_037941895.1	Jeonbuk National University	2022-07-18	ST129	1	4,129,130	29.0	4,129,130	130x
Z1322HCD0006	GCF_037941545.1	Jeonbuk National University	2022-07-12	ST42	3	4,120,221	28.5	4,091,965	151x
Z1322HCD0018	GCF_037941305.1	Jeonbuk National University	2022-07-21	ST42	3	4,169,476	28.5	4,036,161	138x
Z1322HCD0027	GCF_037935125.1	Jeonbuk National University	2022-07-19	ST203	1	4,103,710	28.5	4,103,710	144x
Z1322HCD0031	GCF_037941005.1	Jeonbuk National University	2022-07-30	ST42	5	4,360,013	29.0	4,237,268	145x
Z1322HCD0033	GCF_037934445.1	Jeonbuk National University	2022-08-01	ST129	3	4,289,640	28.5	4,249,241	145x
Z1322HCD0038	GCF_037933585.1	Jeonbuk National University	2022-08-01	ST42	3	4,157,148	28.5	4,116,481	137x
Z1322HCD0041	GCF_037933165.1	Jeonbuk National University	2022-08-03	ST203	1	4,087,117	28.5	4,087,117	156x
Z1322HCD0043	GCF_037932795.1	Jeonbuk National University	2022-09-16	ST17	2	4,119,899	28.5	3,970,152	145x
Z1322HCD0045	GCF_037932505.1	Jeonbuk National University	2022-08-02	ST129	1	4,165,980	29.0	4,165,980	168x
Z1322HCD0061	GCF_037932125.1	Jeonbuk National University	2022-07-28	ST203	1	4,053,455	28.5	4,053,455	153x
Z1322HCD0068	GCF_037931695.1	Jeonbuk National University	2022-08-19	ST129	1	4,115,891	29.0	4,115,891	134x
Z1322HCD0072	GCF_037931105.1	Jeonbuk National University	2022-09-17	ST42	5	4,084,128	28.5	3,937,533	149x
Z1322HCD0073	GCF_037930425.1	Jeonbuk National University	2022-08-24	ST42	3	4,095,038	28.5	4,060,625	179x
Z1322HCD0081	GCF_037930055.1	Jeonbuk National University	2022-08-25	ST203	1	4,121,275	28.5	4,121,275	137x
Z1322HCD0087	GCF_037929775.1	Jeonbuk National University	2022-08-30	ST42	1	4,077,405	28.5	4,077,405	132x
Z1322HCD0092	GCF_037929465.1	Jeonbuk National University	2022-08-31	ST42	1	4,073,072	28.5	4,073,072	147x
Z1322HCD0094	GCF_037929155.1	Jeonbuk National University	2022-08-31	ST203	3	4,142,695	28.5	4,088,385	142x
Z1322HCD0096	GCF_037928795.1	Jeonbuk National University	2022-09-02	ST203	1	4,050,445	28.5	4,050,445	133x
Z1322HCD0097	GCF_037927465.1	Jeonbuk National University	2022-09-02	ST203	2	4,035,169	28.5	3,986,956	162x
Z1322HCD0102	GCF_037926405.1	Jeonbuk National University	2022-09-05	NA	5	4,124,542	28.5	3,814,981	152x
Z1322HCD0107	GCF_037925435.1	Jeonbuk National University	2022-09-08	ST203	3	4,137,496	28.5	4,038,061	146x
Z1322HCD0115	GCF_037924525.1	Jeonbuk National University	2022-09-16	ST203	1	3,951,046	28.5	3,951,046	145x
Z1322HCD0116	GCF_037923795.1	Jeonbuk National University	2022-09-16	ST129	2	4,295,596	29.0	4,287,847	146x
Z1323HCD0018	GCF_037915725.1	Jeonbuk National University	2023-04-11	ST203	1	4,065,075	28.5	4,065,075	143x
Z1323HCD0020	GCF_037915445.1	Jeonbuk National University	2023-04-11	ST42	1	4,255,987	28.5	4,255,987	144x
Z1323HCD0021	GCF_037915205.1	Jeonbuk National University	2023-04-11	ST129	2	4,163,313	29.0	4,149,072	129x
Z1323HCD0032	GCF_037915025.1	Jeonbuk National University	2023-04-12	ST203	1	3,995,145	28.5	3,995,145	157x
Z1323HCD0047	GCF_037914995.1	Jeonbuk National University	2023-04-13	ST42	3	4,127,860	28.5	4,087,080	136x
Z1323HCD0065	GCF_037914985.1	Jeonbuk National University	2023-04-15	ST42	1	4,073,886	28.5	4,073,886	154x
Z1323HCD0085	GCF_037914975.1	Jeonbuk National University	2023-04-17	ST203	1	4,088,406	28.5	4,088,406	146x

NA: Not available

Table S2. Distribution of sequence types and MLST clades in 61 *C. difficile* isolates.

Sequence Type	MLST Clade	Number of Isolates	Relative Frequency (%)
ST17	1	16	26.2
ST1	2	10	16.4
ST2	1	4	6.6
ST42	1	4	6.6
ST8	1	3	4.9
ST35	1	3	4.9
ST37	4	3	4.9
ST54	1	3	4.9
ST100	1	3	4.9
ST3	1	2	3.3
ST11	5	2	3.3
ST203	1	2	3.3
ST14	1	1	1.6
ST48	1	1	1.6
ST51	1	1	1.6
ST55	1	1	1.6
ST129	1	1	1.6
NA	NA	1	1.6

NA: Not available

Table S3. High-impact CdtLoc variants among ST1 and ST11 isolates.

Sequence Type	Sample ID	*cdtR*	*cdtA*	*cdtB*	High-impact CdtLoc variant
ST1	CD9/10/14/24/25/26/28/30/93/150 (*n* = 10)				Not detected
ST11	CD78 (*n* = 1)				Not detected
ST11	CD143 (*n* = 1)	+			*cdtR*: c.322G>T p.Glu108*

Table S4. Distribution of virulence-associated genes in *C. difficile* isolates.

**Sequence Type**	**Sample ID**	***tcdA***	***tcdB***	***fbpA***	***CD0873***	***cbpA***	***CD2831***	***slpA***	***cwp66***	***CD3246***	***cwp84***	***zmp1***	***groEL***
		toxin A	toxin B	adhesin	adhesin	adhesin	adhesin	adhesin	cell wall protein	cell wall protein	cysteine protease	zinc metal-loprotease	chaperonin
ST1	CD9/10/14/24/25/26/28/30/93/150 (*n* = 10)	+	+	+	+		+		+		+	+	+
ST2	CD79/88/141/147 (*n* = 4)	+	+	+	+	+	+			+	+	+	+
ST3	CD17/18 (*n* = 2)	+	+	+	+	+	+		+	+	+	+	+
ST8	CD75/99 (*n* = 2)	+	+	+	+	+	+			+	+	+	+
ST8	CD105 (*n* = 1)	+	+	+		+	+			+	+	+	+
ST11	CD78 (*n* = 1)		+	+	+	+	+			+	+	+	+
ST11	CD143 (*n* = 1)		+	+	+		+		+	+	+	+	+
ST14	CD142 (*n* = 1)	+	+	+	+	+	+		+	+	+	+	+
ST17	CD8/15/76/81/84/94/98/100/101/106/111/115/117/118/119/121 (*n* = 16)	+	+	+	+	+	+		+	+	+	+	+
ST35	CD114/136/137 (*n* = 3)	+	+	+	+	+	+		+	+	+	+	+
ST37	CD12/27/148 (*n* = 3)		+	+	+	+	+	+	+	+	+	+	+
ST42	CD77/80/133/139 (*n* = 4)	+	+	+	+	+	+			+	+	+	+
ST48	CD151 (*n* = 1)			+	+	+	+	+	+	+	+	+	+
ST51	CD19 (*n* = 1)	+	+	+	+	+	+			+	+	+	+
ST54	CD29/109/135 (*n* = 3)	+	+	+	+	+	+		+	+	+	+	+
ST55	CD102 (*n* = 1)	+	+	+	+	+	+		+	+	+	+	+
ST100	CD82/138/145 (*n* = 3)			+	+	+	+		+	+	+	+	+
ST129	CD144 (*n* = 1)	+	+	+	+	+	+		+	+	+	+	+
ST203	CD85/140 (*n* = 2)			+	+	+	+			+	+	+	+
NA	CD83 (*n* = 1)	+	+	+	+	+	+		+	+	+	+	+

NA: Not available

Table S5. AMR gene profiles of *C. difficile* isolates.

Sequence Type	Sample ID	*aac(6′)-aph(2′′)*	*CDD-1*	*CDD-2*	*cplR*	*cfrC*	*nimB*	*erm(52)*	*ermB*	*cdeA*	*dfrF*	*tet(M)*	*tet(40), tet(O/M/O)*	*tetA(P), tetB(P)*
		aminoglycoside	carbapenem	carbapenem	lincosamide; nucleoside; streptogramin	oxazolidinone; lincosamide; streptogramin	nitroimidazole	lincosamide; macrolide; streptogramin	lincosamide; macrolide; streptogramin	fluoroquinolone	diaminopyrimidine	tetracycline	tetracycline	tetracycline
ST1	CD9/10/14/24/25/26/28/30/93 (*n* = 9)			+	+	+	+		+	+				
ST1	CD150 (*n* = 1)			+	+		+			+				
ST2	CD79/88/141/147 (*n* = 4)		+		+		+			+				
ST3	CD17/18 (*n* = 2)	+	+		+		+		+	+				+
ST8	CD75/99 (*n* = 2)		+		+		+			+				
ST8	CD105 (*n* = 1)	+	+		+		+			+				
ST11	CD78 (*n* = 1)		+		+		+			+		+		
ST11	CD143 (*n* = 1)	+	+		+		+			+		+	+	
ST14	CD142 (*n* = 1)		+		+		+			+				
ST17	CD8/15/76/81/84/94/98/100/101/111/115/117/118/119/121 (*n* = 15)	+	+		+		+		+	+				
ST17	CD106 (*n* = 1)		+		+		+		+	+				
ST35	CD114/136/137 (*n* = 3)	+	+		+		+		+	+		+		
ST37	CD12 (*n* = 1)	+		+	+		+		+	+		+		
ST37	CD27 (*n* = 1)	+		+	+	+	+	+	+	+		+		
ST37	CD148 (*n* = 1)	+		+	+		+		+	+	+	+		
ST42	CD77/80/133/139 (*n* = 4)		+		+		+			+				
ST48	CD151 (*n* = 1)	+		+	+		+			+		+		

ST51	CD19 (n = 1)		+		+	+		+	
ST54	CD29/109 (n = 2)	+		+	+	+	+	+	+
ST54	CD135 (n = 1)	+		+	+	+	+	+	
ST55	CD102 (n = 1)			+	+	+		+	
ST100	CD82/138/145 (n = 3)		+		+	+		+	
ST129	CD144 (n = 1)		+		+	+		+	
ST203	CD85/140 (n = 2)		+		+	+		+	
NA	CD83 (n = 1)		+		+	+		+	

NA: Not available

Table S6. Fluoroquinolone resistance-associated *gyrA* and *gyrB* substitution profiles of *C. difficile* isolates.

Sequence Type	Sample ID	*gyrA* p.Thr82Ile	*gyrA* p.Thr82Val	*gyrB* p.Asp426Asn	*gyrB* p.Asp426Val
ST1	CD9/10/14/24/25/26/28/30/93 (*n* = 9)	+	-	-	-
ST1	CD150 (*n* = 1)	-	-	-	-
ST2	CD79/88/141/147 (*n* = 4)	-	-	-	-
ST3	CD17/18 (*n* = 2)	+	-	-	-
ST8	CD75 (*n* = 1)	-	-	-	-
ST8	CD99/105 (*n* = 2)	+	-	-	-
ST11	CD78 (*n* = 1)	-	-	-	-
ST11	CD143 (*n* = 1)	-	-	+	-
ST14	CD142 (*n* = 1)	-	-	-	-
ST17	CD8/15/76/81/84/94/98/100/101/106/111/115/117/118/119/121 (*n* = 16)	+	-	-	-
ST35	CD114/136/137 (*n* = 3)	-	-	-	-
ST37	CD12/27/148 (*n* = 3)	+	-	-	-
ST42	CD77/80 (*n* = 2)	+	-	-	-
ST42	CD133/139 (*n* = 2)	-	-	-	-
ST48	CD151 (*n* = 1)	-	-	-	-
ST51	CD19 (*n* = 1)	-	-	-	-
ST54	CD29/109/135 (*n* = 3)	-	-	-	-
ST55	CD102 (*n* = 1)	-	-	-	-
ST100	CD82/138 (*n* = 2)	-	-	-	-
ST100	CD145 (*n* = 1)	+	-	-	-

**Sequence Type**	**Sample ID**	***gyrA*** **p.Thr82Ile**	***gyrA*** **p.Thr82Val**	***gyrB*** **p.Asp426Asn**	***gyrB*** **p.Asp426Val**
ST129	CD144 (*n* = 1)	-	-	-	-
ST203	CD85/140 (*n* = 2)	-	-	-	-
NA	CD83 (*n* = 1)	-	-	-	-

NA: Not available

Table S7. High-impact variants in functional genes summarized for ST1, ST11, ST17, and ST37 isolates.

Sequence Type	Sample ID	Gene	Nucleotide Change	Amino Acid Change
ST1	CD9/10/14/24/25/26/28/30/93/150 (*n* = 10)	*bclA1*	c.145A>T	p.Lys49*
	CD9/10/14/24/25/26/28/30/93/150 (*n* = 10)	*bclA1*	c.1720C>T	p.Gln574*
	CD9/10/14/24/25/26/28/30/93/150 (*n* = 10)	*flgL*	c.651_653del	p.Asn218del
	CD9/10/14/24/25/26/28/30/93/150 (*n* = 10)	*fliS*	c.349_*1delinsAAAA	p.Ter117LysextTer2
ST11	CD78 (*n* = 1)	*cbpA*	c.1814_1815insGATAAA	p.Asp605delinsGluIleAsn
	CD78 (*n* = 1)	*cbpA*	c.1908_1910del	p.Gly637del
	CD78 (*n* = 1)	*cbpA*	c.2139delinsTTAT	p.Lys713delinsAsnTyr
	CD78 (*n* = 1)	*cbpA*	c.2886_2905delinsCCCGA	p.Gly963_Asp969delinsProAsn
	CD78 (*n* = 1)	*flgL*	c.651_653del	p.Asn218del
	CD78 (*n* = 1)	*fliS*	c.346_*1delinsATAAAAA	p.Leu116_Ter117delinsIleLysIleTer
	CD78 (*n* = 1)	*uvrA*	c.2106_2109delinsTACAA	p.Gln703Thrfs*12
	CD143 (*n* = 1)	*slpA*	c.1901_1908delinsCAGTA	p.Glu634_Val635delinsAla
	CD143 (*n* = 1)	*cbpA*	c.1814_1815insGATAAA	p.Asp605delinsGluIleAsn
	CD143 (*n* = 1)	*cbpA*	c.1908_1910del	p.Gly637del
	CD143 (*n* = 1)	*cbpA*	c.2139delinsTTAT	p.Lys713delinsAsnTyr
	CD143 (*n* = 1)	*cbpA*	c.2886_2905delinsCCCGA	p.Gly963_Asp969delinsProAsn
	CD143 (*n* = 1)	*flgL*	c.651_653del	p.Asn218del
	CD143 (*n* = 1)	*fliS*	c.346_*1delinsATAAAAA	p.Leu116_Ter117delinsIleLysIleTer
ST17	CD8/81/121 (*n* = 3)	*slpA*	c.1901_1908delinsCAGTA	p.Glu634_Val635delinsAla
	CD8/81/121 (*n* = 3)	*flgL*	c.651_653del	p.Asn218del
	CD8/81/121 (*n* = 3)	*fliS*	c.349_*1delinsAAAA	p.Ter117LysextTer2
	CD15/76/84/94/98/100/101/106/111/115/117/118/119 (*n* = 13)	*slpA*	c.1901_1908delinsCAGTA	p.Glu634_Val635delinsAla
	CD15/76/84/94/98/100/101/106/111/115/117/118/119 (*n* = 13)	*fliS*	c.349_*1delinsAAAA	p.Ter117LysextTer2
ST37	CD12/27 (*n* = 2)	*cbpA*	c.3206_3207insCATAGA	p.Asp1069_Ile1070insIleAsp

Table S8. Genomic characteristics and classification of the 24 extrachromosomal elements (ECEs).

Element	Sequence Type	Sample ID	Size (bp)	Core virion modules*	*rep* genes	Closest reference (% identity)	Final classification
CD93_ECE1	ST1	CD93	6670	0/4	1	pJMR5-4 (95.7)	plasmid
CD79_ECE1	ST2	CD79	7180	0/4	0	pCD-S0253 (100.0)	plasmid
CD76_ECE2	ST17	CD76	7639	1/4	0	pCD630 (98.8)	plasmid
CD100_ECE2	ST17	CD100	8329	1/4	0	pCD630 (93.5)	plasmid
CD106_ECE2	ST17	CD106	8041	1/4	0	pCD630 (98.5)	plasmid
CD109_ECE2	ST54	CD109	7793	1/4	0	pCD630 (96.3)	plasmid
CD82_ECE2	ST100	CD82	8104	0/4	1	pCD-ST963 (90.8)	plasmid
CD83_ECE1	NA	CD83	6829	0/4	1	pCD-S0253 (99.2)	plasmid
CD75_ECE1	ST8	CD75	129,338	3/4	1	phiCD38-2 (83.2)	phage-plasmid
CD75_ECE2	ST8	CD75	46,315	3/4	1	phiCD119 (80.4)	phage-plasmid
CD105_ECE1	ST8	CD105	46,181	3/4	1	phiCD119 (88.7)	phage-plasmid
CD82_ECE1	ST100	CD82	110,291	4/4	1	pCD-ST963 (78.2)	phage-plasmid
CD76_ECE1	ST17	CD76	34,286	4/4	0	phiCD119 (94.6)	phage
CD81_ECE1	ST17	CD81	33,486	4/4	0	phiCD119 (92.0)	phage
CD84_ECE1	ST17	CD84	39,279	4/4	0	phiCD38-2 (92.0)	phage
CD94_ECE1	ST17	CD94	33,486	4/4	0	phiCD119 (92.0)	phage
CD98_ECE1	ST17	CD98	33,486	4/4	0	phiCD119 (92.0)	phage
CD100_ECE1	ST17	CD100	33,486	4/4	0	phiCD119 (92.0)	phage
CD106_ECE1	ST17	CD106	33,486	4/4	0	phiCD119 (92.0)	phage
CD114_ECE1	ST35	CD114	134,676	4/4	0	phiCD27 (89.5)	phage
CD77_ECE1	ST42	CD77	32,787	4/4	0	phiCD119 (97.0)	phage
CD80_ECE1	ST42	CD80	32,786	4/4	0	phiCD119 (97.0)	phage
CD109_ECE1	ST54	CD109	34,287	4/4	0	phiCD119 (93.6)	phage
CD85_ECE1	ST203	CD85	33,482	4/4	0	phiCD119 (90.3)	phage

* Core virion modules were defined by the presence of genes encoding capsid, portal, terminase, and tail proteins.

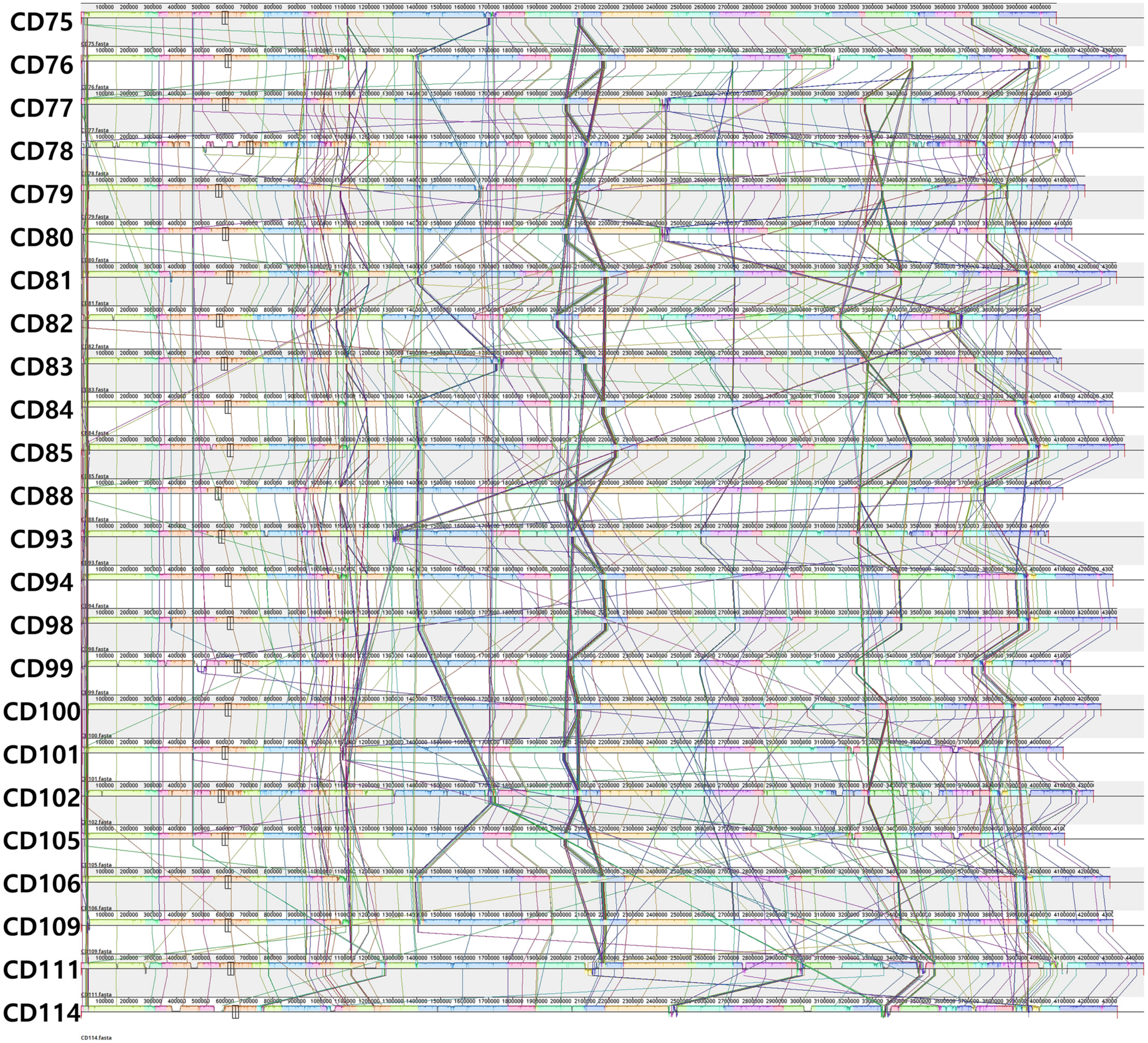

Figure S1. Whole-genome alignment of the 24 hybrid assemblies of *C. difficile* isolates using Mauve (v2.4.0). Colored blocks represent shared homologous regions, and connecting lines mark aligned segments.
